# Clinical efficacy of recombinant human basic fibroblast growth factor combined with ranitidine in the treatment of recurrent oral ulcer and its effect on serum TNF, IL-2 and T-lymphocyte subsets

**DOI:** 10.12669/pjms.40.4.7881

**Published:** 2024

**Authors:** Peng Chen, Bing Liu, Hui Sun

**Affiliations:** 1 Peng Chen, Department of Stomatology, The First Medical Center of Chinese People’s Liberation Army General Hospital, Beijing 100853, China; 2 Bing Liu, Department of Stomatology, Air Force Medical Center of Chinese People’s Liberation Army, Beijing 100142, China; 3 Hui Sun, Department of Diagnostic Radiology, The First Medical Center of Chinese People’s Liberation Army General Hospital, Beijing 100853, China

**Keywords:** Recurrent oral ulcer, Recombinant human basic fibroblast growth factor, Ranitidine, Inflammatory factor, Immune function

## Abstract

**Objective:**

To evaluate the clinical efficacy of recombinant human basic fibroblast growth factor (rh-bFGF) combined with ranitidine in the treatment of recurrent oral ulcer and its effects on serum TNF, IL-2 and T-lymphocyte subsets.

**Methods:**

This was a retrospective study. Eighty patients with oral ulcers admitted to First Medical Center, Chinese PLA General Hospital from July 2021 to June 2022 were randomly divided into the control group and the experimental group (n=40). Patients in the control group were given topical treatment with rh-bFGF gel, while those in the experimental group were given oral treatment combined with ranitidine based on the control group, and both groups were treated continuously for 14 days. The therapeutic effect, pain relief time, ulcer healing time, as well as the differences in the levels of inflammatory factors and T-lymphocyte subsets were compared and analyzed between the two groups.

**Results:**

The overall response rate of the experimental group was 92.5%, while that of the control group was 75%, with a statistically significant difference(P=0.03). After treatment, inflammatory factors indexes in the experimental group were significantly lower than those in the control group, with statistically significant differences (P=0.00). The indexes of T-lymphocyte subsets in the experimental group were significantly higher than those in the control group after treatment, with statistically significant differences (P=0.00).

**Conclusion:**

Recombinant human basic fibroblast growth factor combined with ranitidine is effective in the treatment of recurrent oral ulcers, boasting various benefits such as effectively promoting ulcer healing, reducing pain and inflammatory response, and enhancing immune function.

## INTRODUCTION

Recurrent oral ulcer (ROU), also known as a recurrent aphthous ulcer, is a common oral mucosal disease.^1^ It is commonly found on the margin of the tongue, lips and cheeks, with a prevalence rate of up to 20%.^2^ It is characterized by periodic recurrence and generally has a long initial attack period and a short recurrent attack period. Given the recurrent nature, patients with ROU are affected to varying degrees in terms of feeding and even speech function, seriously affecting their quality of life. The etiology of ROU is so complex that it has not been elucidated yet. The etiologies that have been reported in the literature include microcirculation disturbance, viral and bacterial infection, trace element deficiencies, psychoneurotic factors and genetic factors, etc.^3^ In recent years, abnormal immune function and increased levels of inflammatory factors have been recognized by more and more studies^4^ as important factors involved in the ROU process.

It is believed by Hamedi et al.^5^ that the incidence of ROU has a close bearing on a variety of factors such as abnormal digestive function, impaired immunity and negative emotions. The incidence and recurrence of oral ulcers increase with increasing age as physical function and immune function decline. Although there is no significant threat to patients’ life safety, the disease can pose a great threat to patients’ daily life, such as difficulty in normal communication and severe pain when eating, which reduces the quality of life of patients.^6^ Currently, there are many treatments for ROU in clinical medicine, with priority given to topical medication to relieve pain and accelerate wound healing. Recombinant human basic fibroblast growth factor (rh-bFGF) is beneficial in promoting cell repair and regeneration, while ranitidine, as a potent selective histamine H2 receptor antagonist, can effectively inhibit histamine secretion and promote ulcer healing. In this paper, the clinical efficacy of recombinant human basic fibroblast growth factor combined with ranitidine in the treatment of recurrent oral ulcer was analyzed and its effects on inflammatory factors and T-lymphocyte subsets were investigated.

## METHODS

This was a retrospective study. Eighty patients with oral ulcers admitted to First Medical Center, Chinese PLA General Hospital from July 2021 to June 2022 were randomly divided into two groups: the control group and the experimental group, with 40 cases in each group. According to the data of each indicator in the pre-survey, the sample size is estimated by 95% confidence interval, and the largest one is the sample size of the study. The sample size required for each group was ≥40 cases on the basis of Fisher exact probability. In the experimental group, there were 23 males and 17 females, aged 23-40 years, with an mean age of 44.26±11.73 years, while in the control group, there were 26 males and 14 females, aged 25-63 years, with an mean of 45.08±11.20 years. No significant difference was observed in the comparison of general data between the two groups, which was comparable (Table-I).

**Table-I:** Comparative analysis of the general data of the experimental group and the control group (*χ̅*±*S*) n=40.

Index	Experimental group	Control group	t/ c^2^	P
Age (years)	44.26±11.73	45.08±11.20	0.41	0.68
Male (cases %)	23 (57.5%)	26 (65%)	0.47	0.50
Course of disease (d)	5.47±1.02	5.33±1.17	0.53	0.57
Ulcer diameter (mm)	9.63±0.41	9.57±0.52	0.57	0.32
BMI (kg/m^2^)	23.17±2.31	23.70±2.42	1.00	0.32
***Comorbidities***				
Hypertension1	17 (42.5%)	15 (37.5%)	0.30	0.58
Diabetes mellitus	13 (32.5%)	11 (27.5%)	0.65	0.42
***Poor dietary habits***				
Smoking	15 (37.5%)	18 (45%)	2.12	0.15
Alcohol abuse	9 (22.5%)	11 (27.5%)	2.93	0.10
Spicy food	15 (37.5%)	13 (32.5%)	0.54	0.46

P>0.05.

### Ethical Approval

The study was approved by the Institutional Ethics Committee of First Medical Center, Chinese PLA General Hospital(No.: S2019-396-1; date: March 11, 2019), and written informed consent was obtained from all participants.

### Inclusion criteria:

- Patients meeting the diagnostic criteria for recurrent oral ulcer^7^;
- Patients with an episode frequency ≥3 times/year or a duration of >3 d each time;
- Patients with ulcer healing time >1 week;
- Patients aged 18-65 years;
- Patients without mental illness or cognitive impairment and able to cooperate in completing the study;
- Patients who have not recently used drugs that affect the study results, such as hormone and immunosuppressive drugs;
- Patients who signed the consent form by themselves and their families and were able to cooperate with the completion of the study;
- Patients with complete clinical data.

### Exclusion criteria

- Patients with combined important organ dysfunction such as liver and kidney insufficiency;
- Patients with other pre-existing oral mucosal lesions;
- Patients with concomitant infectious diseases, autoimmune diseases, and peptic ulcers;
- Patients who are allergic to the drugs in this study;
- Patients with a history of taking immunosuppressants and antibiotics in the past one month.

Patients in the control group were given topical treatment with recombinant human basic fibroblast growth factor gel by first performing oral cleansing, especially after debridement of the ulcer, and applying the gel directly to the affected area three times/day. Diet and mouth rinsing were prohibited for 30 minuts after administration. During the treatment period, patients were appropriately supplemented with vitamins and amino acid nutrient solutions, etc. Those in the experimental group were treated orally with ranitidine on the basis of the control group at 0.15g 2/day. Patients in both groups were treated continuously for 14 days.

### Observation indexes

For each patient, ulcer area was measured by using length multiply by width (mm2) at one, two and three weeks after treatment respectively. The degree of pain was scored by Visual Analogue Scale (VAS)(1-10 self-assessment) after treatment at one, two and three weeks respectively

- Comparative analysis of the therapeutic effect of the two groups: clinical efficacy assessment: marked response: reduction of oral ulcer surface >60% after treatment, basically no pain, and oral ulcers show basic healing; moderate response: reduction of oral ulcer surface by 30% to 60% after treatment, significant reduction of pain, occasional attacks of oral ulcers, but with a significant reduction in the number and severity; no response: no significant change or aggravation of oral ulcer symptoms and ulcer surface after treatment. Total effective rate = (marked response + moderate response)/total number of cases × 100%.^8^
- The pain disappearance time and ulcer healing time of the two groups were recorded respectively, and the differences between the two groups were compared and analyzed;
- Comparative analysis of the levels of inflammatory factors: 5ml of peripheral venous blood was drawn from all patients before and in the morning after treatment, respectively, and the levels of tumor necrosis factor (TNF-a), C-reactive protein (CRP), interleukin-6 (IL-6) and other inflammatory factors were detected by enzyme-linked immunosorbent assay (ELISA).
- Comparative analysis of T-lymphocyte subsets: Venous blood was drawn before and after treatment to detect T-lymphocyte subsets such as CD3^+^, CD4^+^, CD8^+^ and CD4^+^/CD8^+^, and the changes of the above indexes were compared and analyzed.

### Statistical analysis

All data in this study were statistically analyzed by SPSS 20.0 software, and measurement data were expressed as(*χ̅*±*S*). The confidence interval is 95%. Two independent sample t-test was used for comparison between groups, paired c^2^ test was used to analyze data within groups, and c^2^ test was used for the comparison of rates. P<0.05 indicates a statistically significant difference.

## RESULTS

The comparative analysis of the therapeutic effects of the two groups is shown in (Table-II). The overall response rate of the experimental group was 92.5%, which was significantly better than 75% in the control group, with a statistically significant difference (P=0.03).

**Table-II:** Comparative analysis of the therapeutic effects of the two groups (*χ̅*±*S*) n=40.

Group	Marked response	Moderate response	No response	Overall response rate
Experimental group	28	9	3	37 (92.5%)
Control group	23	7	10	30 (75%)
c^2^				4.50
P				0.03

P<0.05.

The comparative analysis of pain disappearance time and ulcer healing time between the two groups suggested that the pain relief time and ulcer healing time in the experimental group were significantly shorter than those in the control group, with statistically significant differences (P=0.00) (Table-III).

**Table-III:** Comparative analysis of pain disappearance time and ulcer healing time between the two groups after treatment (*χ̅*±*S*) n=40.

Group	Pain relief time (h)	Ulcer healing time (d)
Experimental group	17.52±5.74	6.38±1.76
Control group	22.47±6.08	8.33±2.09
t	3.74	4.51
P	0.00	0.00

P<0.05.

No statistically significant difference was observed in the comparison of inflammatory factor indices such as TNF-a, CRP and IL-6 between the experimental group and the control group before treatment (P>0.05). After treatment, the indexes of TNF-a, CRP and IL-6 in the experimental group were significantly lower than those in the control group, with a statistically significant difference (P=0.00) (Table-IV).

**Table-IV:** Comparative analysis of changes in NT-proBNP and inflammatory factors in the two groups before and after treatment (*χ̅*±*S*) n=40.

Index		Experimental group	Control group	t	p
CRP (mg/L)	Before treatment	42.33±6.43	42.82±7.04	0.33	0.75
	After treatment*	15.26±6.25	19.57±6.80	2.95	0.00
IL-6 (ng/L)	Before treatment	9.25±1.30	9.43±1.28	0.62	0.53
	After treatment*	4.26±0.73	6.48±1.17	10.18	0.00
TNF-ɑ (ng/L)	Before treatment	38.05±10.86	36.78±11.74	0.50	0.62
	After treatment*	7.89±1.35	12.54±2.16	7.55	0.00

* P<0.05.

No statistically significant difference was observed in the levels of CD3^+^, CD4^+^, CD8^+^ and CD4^+^/CD8^+^ between the two groups before treatment (P>0.05). After treatment, CD3^+^, CD4^+^, CD4^+^/CD8^+^ and other indexes in the experimental group were significantly higher than those in the control group, with a statistically significant difference (P=0.00). There were no significant changes in CD8^+^ in both groups before and after treatment (P>0.05) (Table-V).

**Table-V:** Comparative analysis of T -lymphocyte subsets levels in the two groups before and after treatment (*χ̅*±*S*) n=40.

Index		Experimental group	Control group	t	p
CD3^+^ (%)	Before treatment	42.56±7.24	42.27±7.50	0.18	0.86
	After treatment*	48.27±8.82	43.16±8.75	2.60	0.00
CD4^+^ (%)	Before treatment	23.73±7.28	24.11±7.31	2.23	0.82
	After treatment*	36.80±7.14	31.75±7.26	3.20	0.00
CD8^+^ (%)	Before treatment	23.67±4.52	23.05±4.71	0.61	0.55
	After treatment	24.52±4.06	24.39±4.13	0.14	0.87
CD4^+^/CD8^+^	Before treatment	1.10±0.16	1.12±0.30	0.37	0.71
	After treatment*	1.68±0.14	1.40±0.23	6.57	0.00

* P<0.05.

## DISCUSSION

It was confirmed in our study that the pain relief time and ulcer healing time of the experimental group were significantly shorter than those of the control group (P=0.00); After treatment, TNF-a, CRP, IL-6 and other indexes in the experimental group were significantly reduced compared with the control group (P=0.00). The overall response rate was 92.5% in the experimental group and 75% in the control group, with a statistically significant difference (P=0.03), suggesting the obvious anti-inflammatory and symptom-relieving advantages of recombinant human basic fibroblast growth factor combined with ranitidine. Ranitidine, as an H2 receptor antagonist, has the advantages of long-lasting potency and lower toxic side effects. It can competitively antagonize histamine, inhibit gastric acid secretion, reduce enzyme activity in gastric juice, control the release of inflammatory factors, and avoid the vicious cycle of oral ulcers.^9^ In other words, ranitidine can significantly improve the degree of inflammatory response in patients with ROU and increase the overall response rate of treatment, which is consistent with the findings of Milia et al.^10^ further confirming the good efficacy of ranitidine in the treatment of ROU.

Recurrent oral ulcer (ROU) is a chronic and non-specific oral disease with periodic episodes that are difficult to treat. Histopathologically, it is characterized by localized necrosis, edema and deformation of the oral mucosal epithelium, significant inflammatory cell infiltration in the subepithelial layer, and significant capillary dilation, culminating in the appearance of small, isolated round or oval superficial ulcers in the oral mucosa. Patients with ROU are often accompanied by severe pain, especially after encountering physical stimuli.^11^ The disease may be associated with genetics, infection, mental status, organism immunity, and endocrine disorders, of which abnormal dysregulation of immune and inflammatory factor levels is one of the important ones.^12^ Clinically, topical treatments such as analgesic, anti-inflammatory and accelerated healing, or systemic treatments such as immunomodulators and vitamins, trace elements and corticosteroids are preferentially used to treat ROU, with a view to alleviating body pain, accelerating ulcer healing, prolonging the recurrence interval and reducing the risk of recurrence.^13^

Recombinant human basic fibroblast growth factor, derived from mesodermal and neuroectodermal cells, has a pro-divisive and proliferative effect and can play a role in promoting the formation of neovascularization and wound healing after tissue injury.^14^ It was found that recombinant human basic fibroblast growth factor is derived from adjacent connective tissue and plays a vital role in regulating wound healing.^15^ For one thing, it stimulates tissue production of extracellular matrix, fibroblast-associated protein synthesis, and thus collagen fiber formation.^16^ For another, it accelerates capillary regeneration, ameliorates local blood circulation, and promotes wound healing, which in turn actively accelerates wound repair and improves wound healing quality.^17^

Infiltration of mononuclear cells (large granular lymphocytes and lymphocytes) in the lesion area of ulcers is the earliest histological change of ROU. Some animal experiments have showed that the levels of inflammatory factors such as TNF-α, IL-2, and CRP play a certain role in determining the severity of ROU. 18 TNF-α is a cytokine produced by monocytes, macrophages, mast cells, etc., and its excessive release is the specific process of ulcer disease. Moreover, TNF-α plays a variety of important roles in regulating immune active cells and enhancing the immune function of the body,^19^ such as promoting the secretion of pro-inflammatory factors such as IL-2 and CRP and the release of chemokines such as oxygen free radicals and enhancing the degree of inflammation and oxidative stress response in the body. Also, its mediated inflammatory response can lead to tissue lysis, edema and rupture and other damage to the oral mucosa, with its level positively correlated with the severity of the patient’s condition.^20^

T-lymphocyte subsets interact with each other in the immune system and play a role in maintaining the normal immune function of the body. In case of abnormalities in the number and function of T lymphocyte subsets in the presence of impaired immune function of the body, immune dysfunction will then be caused and infections or autoimmune diseases will be triggered.^21^ According to the findings of Fujisawa et al.^22^, helper T lymphocytes (CD4^+^) are predominantly increased in the pre-ulcer phase of the mouth, while cytotoxic T lymphocytes (CD8^+^) appear significantly increased in the ulcer phase, suggesting that the formation of ulcers is related to the change of T cell subsets. The mechanism may be that abnormal T-lymphocyte immune function causes changes in the body and/or local cellular immune function, and the integrity of oral mucosal epithelium is damaged in patients under the action of certain inductions, leading to the development of oral ulcers. It was confirmed in our study that the indexes such as CD3^+^, CD4^+^, and CD4^+^/CD8^+^ after treatment with recombinant human basic fibroblast growth factor combined with ranitidine were significantly higher than those after recombinant human basic fibroblast growth factor alone, with statistically significant differences (P=0.00). This can be explained mainly by the fact that ranitidine can regulate the T-lymphocyte subset of patients and increase the level of CD3^+^ and CD4^+^ cells, which can enhance the immune function of patients and thus facilitate the regeneration and repair of damaged mucosal epithelial cells, alleviate oral mucosal stress damage and promote wound healing.

### Limitations

The sample size is small, and the effects of psychological factors and other factors on patients are not considered, so the results may be biased to some extent. In addition, the long-term efficacy and recurrence rate of patients are not observed in this study. The sample size will be increased in subsequent studies and multi-center studies will be conducted for further verification.

## CONCLUSION

To put it in a nutshell, recombinant human basic fibroblast growth factor combined with ranitidine is a relatively safe therapy for the treatment of recurrent oral ulcers, leading to various benefits such as significantly reducing the level of inflammatory factors, ameliorating the immune function of patients, reducing the degree of inflammatory response and pain symptoms of patients, and improving clinical efficacy, which is worthy of clinical promotion and application.

### Authors’ Contributions:

**PC** carried out the studies, participated in collecting data, drafted the manuscript, are responsible and accountable for the accuracy and integrity of the work.

**BL** performed the statistical analysis and participated in its design.

**HS** participated in acquisition, analysis, interpretation of data and draft the manuscript.

All authors read and approved the final manuscript.

## References

[1] Yan H, Chen T, Zuo Y, Tu Y, Ai H, Lin Y (2022). A Systematic Review and Meta-Analysis of Acupuncture Treatment for Oral Ulcer. Evid Based Complement Alternat Med.

[2] Hamie L, Hamie M, Kurban M, Abbas O (2022). Eosinophilic ulcer of the oral mucosa:An update on clinicopathologic features, pathogenesis, and management. Int J Dermatol.

[3] Guo X, Zhu T, Yu X, Yi X, Li L, Qu X (2023). Betamethasone-loaded dissolvable microneedle patch for oral ulcer treatment. Colloids Surf B Biointerfaces.

[4] Freitas MO, Fonseca APR, de Aguiar MT, Dias CC, Avelar RL, Sousa FB (2022). Tumor necrosis factor alpha (TNF-α) blockage reduces acute inflammation and delayed wound healing in oral ulcer of rats. Inflammopharmacology.

[5] Hamedi S, Sadeghpour O, Shamsardekani MR, Amin G, Hajighasemali D, Feyzabadi Z (2016). The Most Common Herbs to Cure the Most Common Oral Disease:Stomatitis Recurrent Aphthous Ulcer (RAU). Iran Red Crescent Med J.

[6] Cui J, Lin W, Ma X, Wang J (2022). Clinical Evaluation and Therapeutic Effects of Combination Treatment with Mecobalamin +Vitamin E in Recurrent Oral Ulcer. Clin Ther.

[7] Fitzpatrick SG, Cohen DM, Clark AN (2019). Ulcerated Lesions of the Oral Mucosa:Clinical and Histologic Review. Head Neck Pathol.

[8] Cabras M, Carrozzo M, Gambino A, Broccoletti R, Sciascia S, Baldovino S (2020). Value of colchicine as treatment for recurrent oral ulcers:A systematic review. J Oral Pathol Med.

[9] Giannetti L, Murri Dello Diago A, Lo Muzio L (2018). Recurrent aphtous stomatitis. Minerva Stomatol.

[10] Milia E, Sotgiu MA, Spano G, Filigheddu E, Gallusi G, Campanella V (2022). Recurrent aphthous stomatitis (RAS):guideline for differential diagnosis and management. Eur J Paediatr Dent.

[11] Mumcu G, Karacayli Ü, Yay M, Aksoy A, Taş MN, Armağan B (2019). Oral ulcer activity assessment with the composite index according to different treatment modalities in Behçet's syndrome:a multicentre study. Clin Exp Rheumatol.

[12] Saikaly SK, Saikaly TS, Saikaly LE (2018). Recurrent aphthous ulceration:a review of potential causes and novel treatments. J Dermatolog Treat.

[13] Manfredini M, Guida S, Giovani M, Lippolis N, Spinas E, Farnetani F (2021). Recurrent Aphthous Stomatitis:Treatment and Management. Dermatol Pract Concept.

[14] Liu Y, Liu Y, Deng J, Li W, Nie X (2021). Fibroblast Growth Factor in Diabetic Foot Ulcer:Progress and Therapeutic Prospects. Front Endocrinol (Lausanne).

[15] Freundlich M, Gamba G, Rodriguez-Iturbe B (2021). Fibroblast growth factor 23-Klotho and hypertension:experimental and clinical mechanisms. Pediatr Nephrol.

[16] Agrawal S, Maity S, AlRaawi Z, Al-Ameer M, Kumar TKS (2021). Targeting Drugs Against Fibroblast Growth Factor(s)-Induced Cell Signaling. Curr Drug Targets.

[17] Seitz T, Hellerbrand C (2021). Role of fibroblast growth factor signalling in hepatic fibrosis. Liver Int.

[18] Nakatomi C, Hitomi S, Yamaguchi K, Hsu CC, Harano N, Iwata K (2022). Effect of cisplatin on oral ulcer-induced nociception in rats. Arch Oral Biol.

[19] Surboyo MDC, Boedi RM, Hariyani N, Santosh ABR, Manuaba IBPP, Cecilia PH (2022). The expression of TNF-αin recurrent aphthous stomatitis:A systematic review and meta-analysis. Cytokine.

[20] Liu H, Tan L, Fu G, Chen L, Tan H (2022). Efficacy of Topical Intervention for Recurrent Aphthous Stomatitis:A Network Meta-Analysis. Medicina (Kaunas).

[21] Nakamura M, Oda M, Inoue J, Ito T, Akiba Y, Kitajima M (1995). Effect of basic fibroblast growth factor on reinnervation of gastric microvessels. Possible relevance to ulcer recurrence. Dig Dis Sci.

[22] Fujisawa K, Miyamoto Y, Nagayama M (2003). Basic fibroblast growth factor and epidermal growth factor reverse impaired ulcer healing of the rabbit oral mucosa. J Oral Pathol Med.

